# Glucosinolates in *Brassica* Species: Biosynthesis, Regulation, and Molecular Breeding

**DOI:** 10.3390/ijms27093725

**Published:** 2026-04-22

**Authors:** Shusen Zhao, Mingli Wu, Yanru Chen, Yiyi Xiong, Limei Wang, Hongxun Wang, Maoteng Li

**Affiliations:** 1 College of Life Science and Technology, Wuhan Polytechnic University, Wuhan 430023, China; 20230211005@whpu.edu.cn (S.Z.); wanghongxunhust@163.com (H.W.); 2 Biological Seed Industry Research Institute, Xianghu Laboratory, Hangzhou 311231, China; justuswu@hust.edu.cn; 3 College of Life Science and Technology, Huazhong University of Science and Technology, Wuhan 430074, China; cyrlala1220@163.com (Y.C.); 13995553413@163.com (Y.X.)

**Keywords:** glucosinolates, *Brassica*, transcriptional regulation, molecular breeding, genome editing, secondary metabolism

## Abstract

Glucosinolates (GSLs) are unique sulfur-containing secondary metabolites in *Brassica* crops that critically influence stress resistance, nutritional quality, and economic value. This review systematically summarizes the chemical classification, tissue-specific distribution, and conserved three-phase biosynthetic pathway of GSLs in *Brassica* species. We dissect the core MYB–MYC–WRKY transcriptional regulatory network, elucidate how whole-genome duplication-driven gene functional diversification shapes species-specific GSL accumulation patterns, and outline the multi-layered regulatory system integrating endogenous and exogenous signals. Furthermore, we consolidate recent advances in the genetic dissection of GSL traits and molecular breeding strategies for targeted trait improvement. Finally, we propose a three-tiered regulatory cascade model for GSL metabolism and highlight future research priorities to address current breeding bottlenecks. This work provides a systematic theoretical framework for functional research and precision breeding of GSL metabolism in *Brassica* crops.

## 1. Introduction

**Figure 1:**
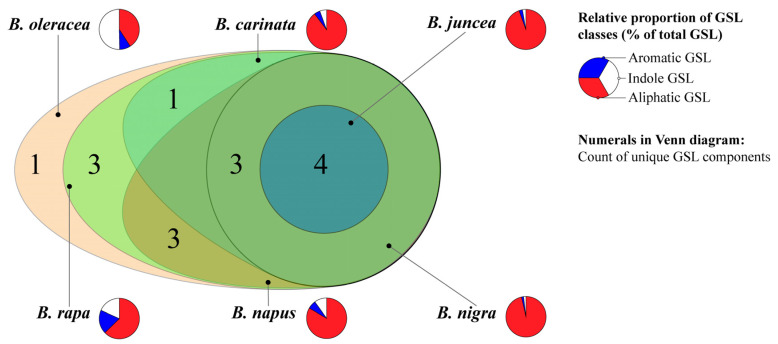
Venn diagram showing the distribution of GSL components among different *Brassica* species. The numerals in the diagram indicate the number of unique GSL components in each respective region. The adjacent equal-size pie charts display the relative proportions of aliphatic (red), aromatic (blue), and indole (white) GSLs in each species.

The genus *Brassica* (*Brassicaceae*) includes many economically important vegetable and oilseed crops, such as Chinese cabbage, cabbage, broccoli, and rapeseed. These crops are rich in characteristic secondary metabolites, especially glucosinolates (GSLs) (Supplementary Table S1)—sulfur-containing compounds unique to the *Brassicales*, which serve as important chemotaxonomic markers [1,2,3,4,5,6], with significant variations in their composition and concentration among different *Brassica* species (Figure 1). GSLs and their hydrolytic products produced by myrosinases or human gut microbiota have dual biological significance: in plants, they act as defense compounds against pathogens and herbivores and contribute to the characteristic flavor of *Brassica* vegetables [7,8]; for humans, dietary GSLs are associated with health benefits including anticarcinogenic and metabolic regulatory effects [9,10,11]. However, excessive accumulation of certain GSLs (e.g., progoitrin) in rapeseed meal can reduce feed quality and cause goiter in livestock [12], underscoring the need for precise regulation of GSL metabolism in crop breeding.

**Figure 2:**
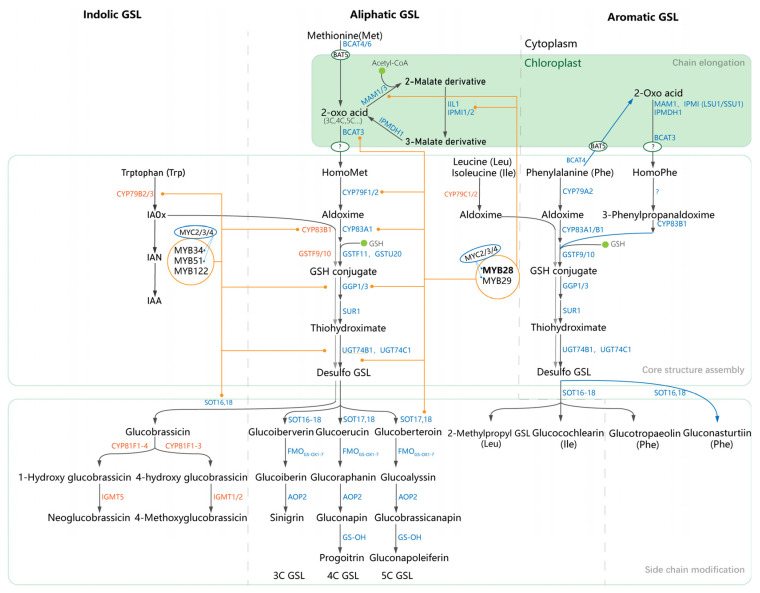
Overview of the biosynthetic pathways for indolic, aliphatic, and aromatic GSLs in *Brassica* species. The gray dashed lines divide the GSL biosynthetic pathway into three major classes: indolic, aliphatic, and aromatic GSLs. The internal boxes partition the entire biosynthetic process into three core stages: chain elongation, core structure assembly, and side chain modification.

Chemically, GSLs are amino acid-derived anionic metabolites with a conserved core structure and diverse side chains [3]. More than 100 GSL structures have been identified. Based on their precursor amino acids and side-chain (R-group) modifications, GSLs are generally divided into three major categories: aliphatic, aromatic, and indole GSLs [8,13]. Their structural diversity is controlled by a complex network involving side-chain elongation, core structure formation, and secondary modifications (Figure 2). Although key genes and regulators have been characterized in model plants, the regulatory landscape in *Brassica* crops remains complex due to widespread genome polyploidization and gene functional divergence.

This review provides a comprehensive summary of the chemical structures, tissue distribution, biosynthesis, and multi-level transcriptional and network regulation of GSLs in *Brassica* species. We further discuss molecular breeding and genetic engineering strategies for modifying GSL traits. By integrating recent advances, this review aims to establish a systematic framework for understanding GSL metabolism and to guide future gene function research and precision breeding of economically important *Brassica* crops.

## 2. Chemical Structures, Types, and Tissue Distribution of GSLs

Different types of GSLs are derived from distinct precursor amino acids: aliphatic GSLs are biosynthesized from alanine, leucine, methionine, isoleucine and valine; aromatic GSLs from phenylalanine and tyrosine; and indolic GSLs from tryptophan [5].

Beyond the diversity imparted by precursor amino acids, the remarkable structural variation in GSLs arises primarily from late-stage side-chain modifications. The R-group can be elongated by one or more methylene units, and both elongated and non-elongated chains may undergo further secondary modifications, including hydroxylation, O-methylation, desaturation, glycosylation, and acylation [7]. For instance, in *Arabidopsis thaliana*, CYP81F family enzymes catalyze the conversion of indol-3-ylmethyl GSL to 4-methoxy-indol-3-ylmethyl GSL, a derivative closely associated with powdery mildew resistance [13]. Acylation can also occur on the GSL core structure, as exemplified by 6′-benzoyl-4-(methylsulfanyl)butyl GSL [7]. Importantly, the biological activities of GSLs are strongly dependent on the structural characteristics of their side chains: sinigrin (aliphatic GSL) from mustard (*B. juncea*) exerts nematicidal and antimicrobial effects, while gluconapin (another aliphatic GSL) from *B. rapa* exhibits prominent antioxidant activity [14], which fully confirms that the structural diversity of GSLs is the core basis for their functional differentiation.

Due to the diversity of side-chain modifications, the number of identified naturally occurring GSL structures in plants has been estimated to range from 88 to 137 to date [4]. Approximately 50 major GSLs have been characterized in edible *Brassica* vegetables, and a single plant species usually accumulates 3 to 4 dominant GSLs, with up to 15 different GSLs detected in individual species in some cases [15]. Bell et al. systematically summarized the composition, abundance, and tissue-specific distribution of GSLs in various *Brassica* vegetables (Supplementary Table S1), showing that aliphatic and indolic GSLs are the dominant subtypes in *Brassica* crops, while aromatic GSLs are generally present at low levels. The most prevalent GSLs in *Brassica* vegetables include aliphatic progoitrin, glucoraphanin, gluconapin, and indolic glucobrassicin [16].

GSLs also exhibit significant tissue-specific accumulation patterns in plants. For example, the aliphatic GSL content in Chinese kale (*Brassica oleracea var. alboglabra*) sprouts is as high as 61.6 mg g^−1^, compared with only 13.7 mg g^−1^ in its leaves. Similarly, broccoli sprouts accumulate much higher GSL concentrations than mature florets [16]. Chen et al. also reported that the content of indol-3-ylmethyl GSL varies markedly among different leaf positions of pak choi (*Brassica rapa* ssp. *chinensis*) [17].

## 3. GSL Biosynthetic Pathway in *Brassica*: Key Steps and Enzymes

Glucosinolate (GSL) biosynthesis is a multi-step, highly conserved process conventionally divided into three core stages (Figure 2): (i) amino acid side-chain elongation, (ii) core GSL structure formation, and (iii) secondary modification of the side chain [5]. This complex pathway is governed by an intricate network of structural genes and transcription factors. Although the pathway was initially elucidated in the model plant *Arabidopsis thaliana* [5], subsequent studies have confirmed the evolutionary and functional conservation of key GSL biosynthetic genes (e.g., *CYP79F1* [18], *AOP2* [19,20], *CYP83F1* [21], *MAM1* [21], *SOT* [22]) and regulatory transcription factors (e.g., MYB28 [18,23,24], MYB29 [25], MYB51 [26]) in *Brassica* species. The lineage-specific characteristics and diversification of GSL structural genes in polyploid *Brassica* will be addressed in detail in Section 5 and subsequent sections. The present section provides a concise overview of the biosynthetic pathway, drawing primarily on examples from *Arabidopsis* to illustrate the roles of key enzymes involved in GSL biosynthesis.

The side-chain elongation stage primarily acts on methionine-derived aliphatic GSLs, while phenylalanine-derived aromatic GSLs typically bypass this step [5]. This process involves a cyclic reaction: deamination of amino acids catalyzed by BCAT4/6, carbon addition via α-keto acid conversion mediated by MAM1/2/3 and IPMDH1, and re-amination catalyzed by BCAT3, which can add up to nine carbon atoms to generate diverse homo-methionine precursors [8].

The core GSL structure formation is a highly conserved pathway for all GSL classes. Side-chain elongated or unmodified amino acid precursors are first converted to aldoximes by CYP79 enzymes, followed by further processing by CYP83 enzymes, glutathione conjugation via GSTs, and formation of thiohydroximate intermediates. The final steps of core structure assembly include glycosylation by UGT74B1 and sulfation catalyzed by SOT16/17/18 [7,27].

The secondary modification stage is the main driver of GSL structural diversity, through post-synthetic modifications of the side chain. Key enzymes in this stage include FMO_GS-OX_1–7, which catalyze the oxidation of methylthioalkyl GSLs to methylsulfinylalkyl GSLs, and AOP2, which mediates hydroxylation to generate compounds such as progoitrin. Indolic GSLs (e.g., glucobrassicin) also undergo specific sulfation and methylation reactions in this stage [3,13]. The coordinated expression of these structural genes is orchestrated by a complex transcriptional regulatory network, which is the focus of the next section.

## 4. Key Transcription Factor Families (MYB, MYC, WRKY) Regulating GSL Biosynthesis

The multi-stages GSL biosynthetic pathway is subject to precise, hierarchical transcriptional regulation, which is predominantly governed by three conserved core transcription factor (TF) families in *Arabidopsis thaliana* and *Brassica* relatives: R2R3-MYB, basic helix–loop–helix (bHLH, mainly MYC family), and WRKY families. These TFs form the core regulatory module of GSL metabolism, directly targeting the promoters of structural genes in the biosynthetic pathway to control GSL production, and their functional framework is highly conserved across *Brassicales* [28]. Similarly, this section illustrates the functions of key transcription factors using examples primarily drawn from *Arabidopsis*. The lineage-specific functional diversification of these regulators in *Brassica* species will be examined in detail in Section 5 and thereafter.

### 4.1. R2R3-MYB Transcription Factors: The Master Switches for Class-Specific GSL Biosynthesis

**Figure 3:**
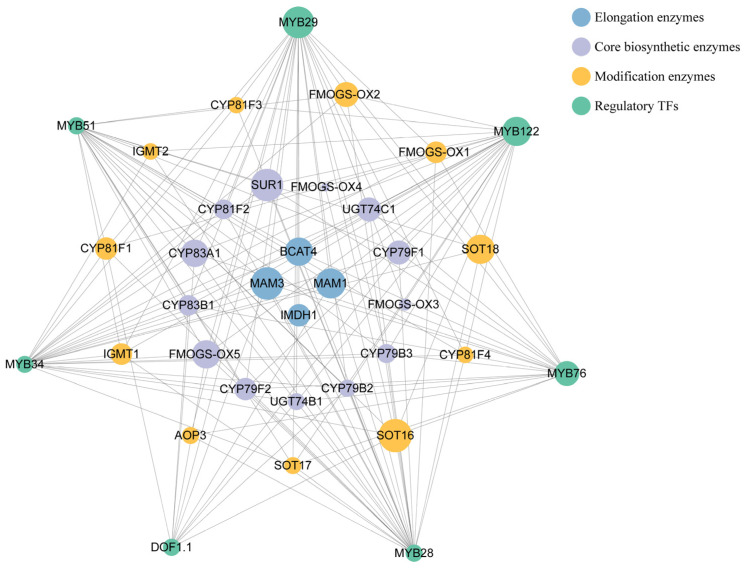
Protein–protein interaction (PPI) network of GSL biosynthesis-related genes and their regulatory transcription factors in *Brassica*. Nodes are color-coded according to their functional roles, organized from the innermost to outermost layer: blue (side-chain elongation enzymes), purple (core biosynthetic enzymes), orange (side-chain modification enzymes), and green (regulatory transcription factors, TFs). Node size is proportional to node degree (the number of direct connections for each node). To improve visual clarity and highlight core regulatory relationships, interaction edges between transcription factors are omitted in this figure.

In *Brassica*, R2R3-MYB TFs constitute the key determinants of GSL biosynthesis (Figure 3), with two well-characterized submodules specifically governing the two dominant GSL branches (aliphatic and indolic GSLs), while the regulatory mechanism for aromatic GSLs remains to be fully elucidated. These MYBs directly bind to the conserved motifs in the promoters of structural genes in all three stages of GSL biosynthesis, activating their transcription and determining the class and abundance of final GSL products.

The aliphatic GSL pathway is specifically regulated by R2R3-MYB subgroup 12 members: MYB28 (HAG1) and MYB29 (HAG3). These TFs directly activate the expression of multiple genes involved in methionine side-chain elongation (including *MAM1/MAM3*, *BCAT3*, *IPMI*) and core structure formation (including *CYP79F1/F2*, *CYP83A1*, *UGT74B1*, *SOT18*) [13,29,30]. Among them, MYB28 acts as the primary dominant regulator, with the *myb28* single mutant showing a reduction in total leaf aliphatic GSLs to approximately 44% of wild-type (Col-0) levels, while MYB29 provides synergistic and compensatory activity, with the *myb29* single mutant only reducing total leaf aliphatic GSLs to around 92% of wild-type levels. Notably, the *myb28 myb29* double mutant exhibits a near-complete loss of aliphatic GSLs, confirming their non-redundant core roles in aliphatic GSL biosynthesis [13,31]. Additionally, a metabolite-mediated feedback loop has been identified: the secondary modification enzyme AOP2 can increase the expression of *MYB28* and *MYB29*, thereby modulating the type and abundance of aliphatic GSLs [32].

The indolic GSL pathway, derived from tryptophan, is preferentially controlled by another set of R2R3-MYB subgroup 12 members: MYB34 (ATR1), MYB51, and MYB122. MYB34 was first identified as a transcriptional activator of the core indolic GSL biosynthetic genes *CYP79B2* and *CYP79B3* [33]. Subsequent studies have confirmed that MYB51 and MYB122 also strongly co-express with and activate a suite of indolic GSL-related genes, including *CYP79B2/B3*, *CYP83B1*, *CYP81F2*, and *IGMT1/2* [29,34]. These three MYBs exhibit clear functional divergence in tissue specificity, enabling spatiotemporal regulation of indolic GSL accumulation across different plant tissues [34].

### 4.2. The MYC-MYB Regulatory Module: Core Transcriptional Complex for GSL Biosynthesis

MYB regulators do not act in isolation; rather, they function in concert with bHLH transcription factors of the MYC family (MYC2, MYC3, MYC4, and MYC5) to form the core MYC–MYB transcriptional complex, which is essential for activating GSL biosynthetic genes. These MYC TFs directly interact with R2R3-MYB TFs—including aliphatic-related MYB28/MYB29 and indolic-related MYB34/MYB51—and the formation of MYC–MYB complexes markedly enhances both the DNA-binding affinity and transcriptional activation capacity of the MYB regulators on their target genes [28,35].

Genetic evidence has confirmed the indispensable role of MYC TFs in GSL regulation: the *myc2/3/4* triple mutant shows a 70–80% reduction in basal aliphatic GSL content compared to wild-type plants, and completely loses jasmonic acid (JA)-induced GSL accumulation, with no detectable increase in either aliphatic or indolic GSL levels following JA treatment [35]. This MYC-MYB module is functionally conserved across *Brassicales*, serving as the core protein complex that executes the transcriptional regulation of GSL biosynthesis [7,13].

### 4.3. WRKY Transcription Factors: Specific Regulators of Indolic GSL Biosynthesis

Beyond the core MYC-MYB module, WRKY TFs provide an additional layer of specific regulation for GSL biosynthesis, particularly for the indolic GSL branch. The most well-characterized member is WRKY33, which has been identified as a critical regulator of indolic GSL secondary modification [36]. WRKY33 directly binds to the W-box motifs in the promoters of indolic GSL secondary modification genes, including *CYP81F2* and *IGMT1/2*, and activates their transcription to promote the accumulation of modified indolic GSLs [34,36].

In contrast to the broad regulatory scope of the MYC-MYB module (covering both aliphatic and indolic GSLs), WRKY33-mediated regulation represents a branch-specific regulatory pathway that preferentially controls indolic GSL biosynthesis [36]. This regulatory pattern enables independent fine-tuning of indolic GSL accumulation without affecting the aliphatic GSL pool, thereby enhancing the flexibility of the GSL transcriptional regulatory network.

In summary, GSL biosynthesis is governed by a conserved, hierarchical transcriptional regulatory system: R2R3-MYB factors act as the class-specific key determinants to determine the direction of GSL metabolism, MYC-bHLH factors form core complexes with MYBs to execute transcriptional activation, and WRKY factors provide branch-specific regulatory plasticity for indolic GSLs (Supplementary Figure S1). While this functional framework is well established in the model plant *Arabidopsis*, the lineage-specific diversification of these core regulators in polyploid *Brassica* species will be systematically elaborated in the following section.

## 5. Whole-Genome Duplication-Driven Gene Diversification Regulates GSLs in *Brassica* Species

Unlike the diploid model plant *Arabidopsis thaliana*, *Brassica* species have undergone extensive whole-genome duplication (WGD) and lineage-specific triplication events, resulting in a complex polyploid evolutionary history that underpins their highly divergent GSL profiles [37,38]. This widespread genetic expansion has driven gene loss, sub-functionalization, and neo-functionalization of both the core regulatory TFs and GSL biosynthetic structural genes, which together directly shape the species- and cultivar-specific GSL accumulation patterns observed across cultivated *Brassica* crops.

### 5.1. Diversification of Core GSL Regulatory Transcription Factors in Brassica Species

**Figure 4:**
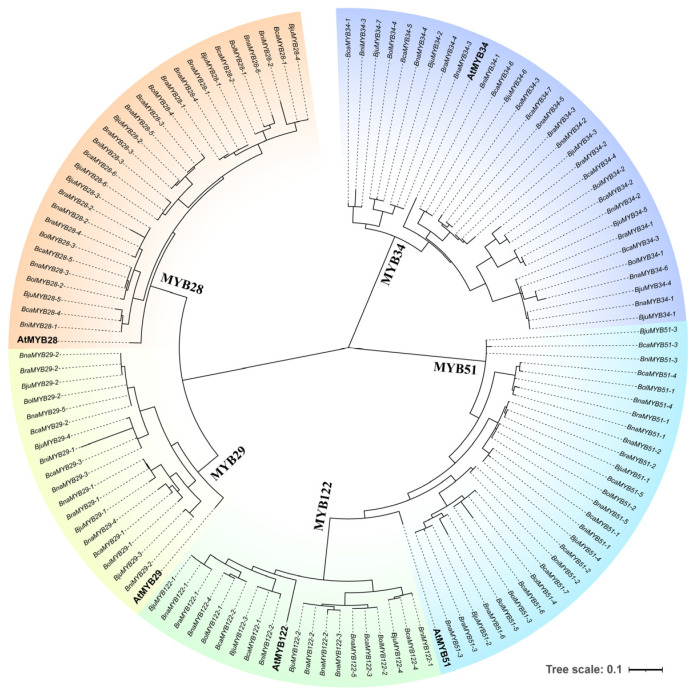
Phylogenetic tree of the MYB28/29/34/51/122 TFs family in *Brassica* species and *Arabidopsis thaliana*. This phylogenetic tree was constructed using the Maximum Likelihood (ML) method with 1000 bootstrap replicates. The tree scale (0.1) represents genetic distance. The prefixes of gene names correspond to species: At (*Arabidopsis thaliana*), Bju (*Brassica juncea*), Bni (*Brassica nigra*), Bra (*Brassica rapa*), Bna (*Brassica napus*), Bol (*Brassica oleracea*), and Bca (*Brassica carinata*). Gene data for the phylogenetic tree are provided in Supplementary Table S2.

**Figure 5:**
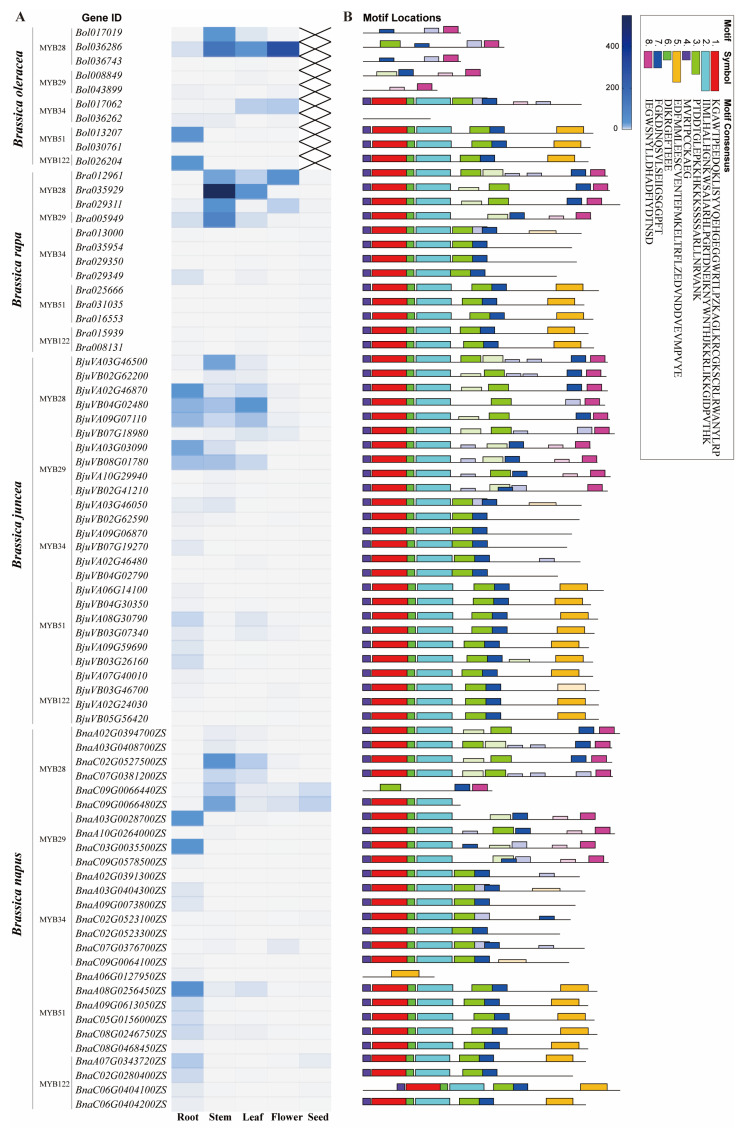
Tissue expression patterns and conserved motif structures of MYB transcription factors involved in GSL biosynthesis regulation across different *Brassica* species. (**A**): Heatmap showing the expression levels of MYB family transcription factors in different tissues. The color intensity is positively correlated with the expression level, and “×” indicates no expression data available for the corresponding gene in that tissue. (**B**): Conserved motif distribution of MYB family transcription factors predicted by MEME analysis. Gene expression levels and motif prediction data are shown in Supplementary Table S3.

The most notable diversification induced by WGD events occurs in the core regulatory TF families of GSL biosynthesis, particularly the R2R3-MYB family—the key determinants of GSL metabolism—which exhibits significant copy number variation and functional divergence in *Brassica* species. As shown in the phylogenetic tree (Figure 4), the MYB family has undergone extensive expansion following WGD, while the expression heatmap (Figure 5) reveals distinct tissue-specific expression patterns across different organs. Among these, the expression data for *B. rapa* in Figure 5 were derived from a previous study [39].

For example, multiple copies of *BjuMYB28* in the allotetraploid *B. juncea* show overlapping yet distinct regulatory roles in aliphatic GSL biosynthesis. Among the four identified *BjuMYB28* homologs, *BjuMYB28-1* and *BjuMYB28-2* are predominantly expressed in seeds and act as the major determinants of seed aliphatic GSL content. Silencing either *BjuMYB28-1* or *BjuMYB28-2* alone results in only a partial reduction in seed GSLs, whereas simultaneous silencing of both leads to a dramatic decrease in seed GSL content to 11.26 μmol g^−1^ DW, which is well below the feed safety threshold of <30 μmol g^−1^ DW [23]. This functional divergence of *BjuMYB28* copies provides a genetic basis for precise metabolic engineering, as demonstrated by the suppression of the *GSL-ALK* gene family, which enriches the anti-cancer compound glucoraphanin up to 43.11 μmol g^−1^ DW in *B. juncea* seeds [37].

*BnaC2.MYB28* has been identified as the major determinant of seed GSL content in *Brassica napus*, and its functional importance has been validated by CRISPR/Cas9-mediated gene editing. Specifically, knockout of *BnaC2.MYB28* in the high-GSL parent G120 (33.0 ± 2.4 μmol g^−1^) reduced seed GSL content to ~14–17 μmol g^−1^, which is even lower than the low-GSL parent ZY50 (17.7 ± 1.5 μmol g^−1^). Conversely, overexpression of *BnaC2.MYB28* in ZY50 led to a dramatic increase, with one transgenic line reaching 161.3 ± 15.8 μmol g^−1^ [24,40]. Different *BnMYB28* homologous genes also exhibit distinct tissue-specific expression patterns: *BnA9.MYB28* and *BnC9.MYB28* are expressed in nearly all tissues analyzed, whereas *BnC7.MYB28* shows no detectable expression in any tissue tested. *BnC9.MYB28* has the highest expression level in siliques compared to buds, stems, and petals, while *BnA9.MYB28* shows peak expression in cauline leaves, siliques, and buds [41]. However, this functional divergence is not merely a case of simple redundancy or subfunctionalization. Recent studies have revealed a more nuanced and contentious picture. For instance, the predominance of specific *MYB28* homologs appears to depend on genetic background: *BnaA9.MYB28* acts as a major regulator in European winter-type rapeseed, whereas *BnaC2.MYB28* exerts a stronger effect in Asian semi-winter types [42]. Furthermore, compelling evidence from Tu et al. indicates that *BnaC09.MYB28* regulates GSL content in both seeds and leaves, whereas downregulation of *BnaA02.MYB28* or *BnaC02.MYB28* is expected to reduce seed GSL without a concomitant decrease in leaves [43]. This genetic divergence and tissue-specific expression pattern directly dictate the species-specific aliphatic GSL accumulation profiles, which are critical for the trait differentiation between oilseed and vegetable *Brassica* crops [44].

The complexity within the MYB family extends beyond MYB28. While MYB29 is generally considered a synergistic partner to MYB28 in aliphatic GSL regulation, this view is overly simplistic in polyploid *Brassica*. A striking example in allotetraploid *B. juncea* reveals that among its four *MYB29* homologs, *BjuA03.MYB29* suppresses indolic GSL accumulation, whereas *BjuA10.MYB29* enhances it, even though both function identically in activating aliphatic GSL biosynthesis [45]. This coexistence of synergistic and antagonistic functions among homologs of the very same transcription factor underscores that the regulatory logic in polyploids cannot be captured by simple “redundancy” models.

In addition to MYB TFs, the MYC and WRKY family members also exhibit copy number variation and functional diversification in *Brassica* species (Supplementary Figures S2–S5;). Multiple homologous copies of *MYC2* and *WRKY33* have been identified in *B. napus*, *B. rapa*, and *B. oleracea*, with distinct expression patterns in response to biotic and abiotic stresses, which contribute to the species-specific stress-induced GSL accumulation in *Brassica* crops [37,46]. This diversification of core regulatory TFs provides *Brassica* species with a more flexible and complex regulatory system for GSL metabolism compared to *Arabidopsis*.

### 5.2. Diversification of GSL Biosynthetic Structural Genes in Brassica Species

Beyond the regulatory TFs, the structural genes involved in GSL biosynthesis also exhibit extensive polymorphism and copy number variation in *Brassica* species, which modulates the regulatory output of the upstream TF network and further expands GSL structural diversity. For the side-chain elongation stage of aliphatic GSL biosynthesis, polymorphisms in the MAM gene cluster are crucial for determining the final chain length of GSL products; silencing *MAM* genes in *B. napus* via RNAi dramatically shifts the GSL product distribution, leading to the elimination of C5 aliphatic GSLs in individual transgenic lines and an approximate five-fold (80%) reduction in C4 aliphatic GSLs in extreme cases. Meanwhile, this silencing event induces the de novo accumulation of C3-type 2-propenyl GSL—an aliphatic GSL undetectable in wild-type *B. napus*—with its content ranging from 0.07 to 1.88 μmol g^−1^ seed in transgenic canola lines and rising up to 4.45 μmol g^−1^ seed (accounting for 40–50% of total aliphatic GSLs) in the high-GSL progenies derived from crosses of transgenic canola and high-GSL rapeseed [47].

In the secondary modification stage, the *AOP2* gene, which converts the nutritionally beneficial glucoraphanin into the undesirable progoitrin, often undergoes natural functional inactivation in *Brassica* vegetables (e.g., broccoli) and is a key target for gene editing to enhance crop nutritional value [48]. Suppression of *AOP2* (*GSL-ALK*) family members in *B. juncea* via intron-spliced hairpin RNAi has been shown to enhance glucoraphanin accumulation—reaching up to 43.11 μmol g^−1^ dry weight (DW) in transgenic seeds, compared to only trace levels in wild-type plants—and to improve crop disease resistance, as evidenced by significantly reduced lesion development upon inoculation with the fungal pathogen *Sclerotinia sclerotiorum* [37]. Additionally, enzymes involved in the late stages of GSL biosynthesis, such as CYP79F1 (which specifically regulates C3-type GSL accumulation) and members of the FMO_GS-OX_/SOT families, also exhibit extensive copy number variation across *Brassica* species [18,49,50,51]. This genetic variation enables precise and diverse fine-tuning of GSL structures, further expanding the regulatory potential of the upstream TF network and contributing to the unique GSL profile of each *Brassica* species.

In summary, the high complexity and diversity of GSL profiles in *Brassica* species are a direct consequence of their polyploid genome structure driven by WGD events. The co-diversification of core regulatory TFs and their target GSL biosynthetic structural genes through gene loss, sub-functionalization, and neo-functionalization not only distinguishes *Brassica* species from the model plant *Arabidopsis* but also lays the fundamental genetic basis for the formation of multi-layered, coordinated GSL regulatory networks. This diversification provides the evolutionary flexibility for *Brassica* species to integrate nutrient, hormonal, and environmental signals to fine-tune GSL metabolism, which will be systematically elaborated in the following chapter.

## 6. Coordinated Regulatory Networks of GSLs: Integration of Hormonal, Nutritional, and Environmental Signals in *Brassica* Species

Building on the regulatory framework established in the preceding chapters, GSL biosynthesis in *Brassica* species is subject to further modulation by a complex network that integrates endogenous hormonal signals, nutrient availability, and external environmental stresses. This multi-layered regulatory system connects the core transcriptional machinery with broader physiological and developmental programs, thereby fine-tuning GSL composition and abundance to balance growth, defense, and nutritional quality under fluctuating environmental conditions.

### 6.1. Hormonal Signal Integration in GSL Regulatory Networks

Hormonal signals are the core endogenous regulators that link plant development and stress responses with GSL biosynthesis, predominantly acting through the core MYC-MYB transcriptional module established. Jasmonic acid (JA), the primary defense hormone regulating GSL metabolism, is mediated by the conserved COI 1–JAZ–MYC cascade. In the absence of JA, JAZ proteins bind to MYC TFs and block their interaction with MYB regulators, thereby repressing GSL biosynthesis. Upon herbivore or pathogen attack, JA is rapidly synthesized, leading to the ubiquitination and degradation of JAZ proteins, the release of activated MYC TFs, and the formation of MYC-MYB complexes that trigger a rapid upregulation of GSL biosynthetic genes [52,53]. This JA-MYC-MYB cascade is functionally conserved in *Brassica* crops, and its rapid activation upon JA treatment or herbivore feeding establishes an efficient chemical defense barrier [7,13].

Beyond JA, multiple other hormones participate in the coordinated regulation of GSL biosynthesis through crosstalk with the core transcriptional module. Abscisic acid (ABA) cooperates with JA under abiotic stress to upregulate the expression of *MYB34* and its downstream indolic GSL biosynthetic genes [33,34]. Salicylic acid (SA) and ethylene (ET), which are core hormones in pathogen resistance, interact with JA signaling to specifically induce *MYB51* and *WRKY33* expression, thereby promoting the accumulation of pathogen-inducible indolic GSLs [34,36]. This hormonal crosstalk enables the precise integration of multiple endogenous developmental signals into the core GSL regulatory network.

### 6.2. Nutrient Availability Modulates GSL Metabolic Balance

Nutrient availability is a critical determinant of GSL biosynthesis, as GSLs are nitrogen- and sulfur-containing secondary metabolites, and their production is tightly coupled with plant primary nutrient metabolism. Sulfur nutrition is the most prominent regulatory factor: under sulfur deficiency, the central transcription factor SLIM1 (sulfur limitation 1) acts as a negative regulator of indolic GSL accumulation in roots; it represses the expression of GSL biosynthetic genes while inducing the transcription of sulfate assimilation genes (e.g., *APS*, *APR*), thereby prioritizing primary sulfur metabolism to maintain basal plant growth [54,55]. This transcriptional reprogramming is mediated by the crosstalk between SLIM1 and core R2R3-MYB factors (e.g., MYB28, MYB34), which together coordinate the balance between primary sulfur assimilation and secondary GSL biosynthesis [56].

Carbon and nitrogen nutrition also play pivotal roles in modulating GSL metabolism, often mediating the trade-off between plant growth and defense. Carbohydrate signaling (e.g., glucose) promotes indolic GSL accumulation, coupling primary carbon metabolism with secondary defense responses through the synergistic regulation of MYB TFs and bZIP transcription factors [57]. Nitrogen availability, by contrast, often reshapes GSL profiles: high nitrogen levels favor vegetative growth over GSL biosynthesis, leading to reduced GSL accumulation in most *Brassica* crops [7,13]. This nutrient-mediated regulation ensures that GSL biosynthesis is dynamically adjusted according to the plant’s nutritional status, avoiding excessive metabolic costs.

### 6.3. Environmental Stress Signals Shape GSL Profiles in Brassica Crops

External environmental stresses further fine-tune GSL accumulation through the coordinated regulatory network, predominantly by modulating hormonal signaling pathways and the activity of core TFs. Abiotic stresses, including salinity, heavy metals, and low temperature, typically alter the signaling pathways of JA, ABA, and reactive oxygen species (ROS); these signaling changes indirectly reshape GSL composition and abundance via the core MYB, MYC, and WRKY regulatory networks [7,13]. For example, salt stress induces ABA accumulation in *Brassica* crops, which upregulates *MYB34* expression and promotes indolic GSL accumulation to enhance stress tolerance. In Chinese cabbage (*Brassica rapa*), salt stress significantly upregulates BrMYB34 expression by 2.8–4.2 fold, accompanied by increased indolic GSL contents. [34]. Light signals also modulate the protein stability of MYC TFs, further linking photomorphogenesis with GSL secondary metabolism [35].

Biotic stresses, including pathogen infection and herbivore attack, trigger the rapid activation of the JA-MYC-MYB cascade and WRKY33-mediated regulatory pathway, leading to the targeted accumulation of defensive GSLs [36,53]. Notably, the WGD-driven gene diversification in *Brassica* species provides enhanced regulatory plasticity in response to environmental stresses: the redundant copies of core TFs and structural genes enable *Brassica* crops to tailor their GSL profiles against specific biotic or abiotic threats, enhancing their adaptive capacity to fluctuating environments [37,38].

### 6.4. Auxiliary Regulators and Epigenetic Modifications Add Regulatory Complexity

Auxiliary transcriptional regulators and epigenetic modifications further enrich the multi-layered GSL regulatory network, providing additional fine-tuning beyond the core TF families. The DOF transcription factor AtDof1.1 (also known as OBP2) positively regulates indolic GSL biosynthesis by forming a positive feedback loop with MYB34 and MYB51, which amplifies JA-mediated and mechanical wounding-induced GSL accumulation [29,58]. The calmodulin-binding nuclear protein IQD1 integrates Ca^2+^ signaling into the GSL regulatory network, enhancing both GSL levels and plant resistance to biotic and abiotic stresses [59].

Additionally, epigenetic regulation represents an important, yet less characterized, layer of GSL metabolic control. The HP1-like protein TU8 has been implicated in the epigenetic regulation of plant secondary metabolism, with mutations in *TU8* leading to altered GSL accumulation via chromatin state modifications [60]. This epigenetic regulation enables the heritable fine-tuning of GSL profiles in *Brassica* crops, providing a new direction for GSL trait improvement.

Notably, the polyploidization history of *Brassica* species has created extensive gene redundancy in this coordinated regulatory network. This genetic buffer provides unique evolutionary flexibility, facilitating the sub-functionalization and neo-functionalization of regulatory elements, and offers a valuable opportunity to target specific gene copies to resolve the inherent “quality-defense trade-off” in *Brassica* crops—i.e., balancing nutritional quality (e.g., low progoitrin content) and defensive capacity (e.g., high beneficial GSL levels). Collectively, the coordinated regulatory networks described herein establish a theoretical framework for the precision molecular breeding strategies aimed at optimizing GSL traits, which will be elaborated in the following chapter.

## 7. Molecular Breeding and Genetic Engineering Strategies for GSL Traits in *Brassica* Species

As elaborated in previous chapters, GSLs are central determinants of defensive capacity, nutritional quality, and flavor in *Brassica* crops. A comprehensive understanding of their biosynthesis and regulation now provides a solid foundation for the targeted improvement of GSL-related traits. Breeding objectives in *Brassica* are highly crop type-specific: for oilseed crops (*B. napus*, *B. juncea*), the primary goal is to reduce antinutritional GSLs (especially progoitrin) in seeds to enhance the feeding value of rapeseed meal, while ideally retaining defensive GSLs in vegetative tissues to maintain pest and disease resistance. Conversely, for vegetable-type crops, the focus is on enriching health-promoting GSLs (notably glucoraphanin) in edible tissues while minimizing bitter or undesirable components [7,61]. The recent proliferation of high-quality *Brassica* genome assemblies, multi-omics technologies, and precision gene-editing tools has enabled the functional validation of key metabolic and regulatory nodes, thereby accelerating the transition from conventional breeding to precision molecular improvement of GSL traits [13].

### 7.1. Breeding Objectives and Genetic Basis of GSL Traits in Brassica Crops

GSL content and composition are typical quantitative traits controlled by multiple genes, with core genetic determinants falling into three categories: (1) structural genes involved in side-chain elongation and core structure formation (e.g., *MAM*, *BCAT*, *CYP79*, *CYP83*); (2) side-chain modification genes (e.g., *AOP*, *FMO_GS-OX_*); (3) upstream transcriptional regulators (e.g., *MYB28/MYB29*, *MYB34/MYB51/MYB122*) [13]. As elaborated above, multiple rounds of WGD in *Brassica* species have led to the extensive expansion of GSL-related genes, with approximately 300 GSL-associated genes identified in *B. napus* alone, most of which exhibit highly differentiated tissue-specific expression patterns [62].

This unique polyploidization history creates both challenges and opportunities for GSL trait breeding. On one hand, functional redundancy of homologous genes increases the difficulty of complete trait modification; on the other hand, the subfunctionalization and neofunctionalization of duplicated genes enable the precise manipulation of tissue-specific GSL profiles without compromising overall plant fitness [62]. This evolutionary feature forms the core theoretical basis for the precision breeding strategies discussed in this chapter. Analyses of natural populations and biparental segregating populations have further revealed that quantitative trait loci (QTLs) for GSL traits often colocalize with biosynthetic gene clusters or major regulatory genes. For example, loci corresponding to *AOP*, *MAM*, and *CYP79F* are tightly associated with aliphatic GSL side-chain elongation and modification [32,63,64], whereas allelic variation in master regulators such as *MYB28/MYB29* markedly affects total GSL levels and flux distribution among different pathway branches [13,37,38]. These findings provide valuable targets for marker-assisted selection (MAS) and genomic selection (GS) in *Brassica* breeding.

### 7.2. QTL/GWAS-Based Genetic Dissection and Marker-Assisted Breeding of GSL Traits

The genetic dissection of GSL accumulation in *Brassica* species has evolved from low-resolution bi-parental QTL mapping to high-resolution genome-wide association studies (GWAS) and pan-genome-based structural variation (SV) analysis. Among these approaches, the integrated strategy coupling QTL mapping with transcriptomics has been widely recognized as a robust and efficient paradigm for decoding the genetic architecture of secondary metabolites in allopolyploid *Brassica napus*. This approach enables precise identification of QTL hotspots, rapid prioritization of causal genes, and thus greatly accelerates the development of functional markers for GSL traits [65].

**Figure 6:**
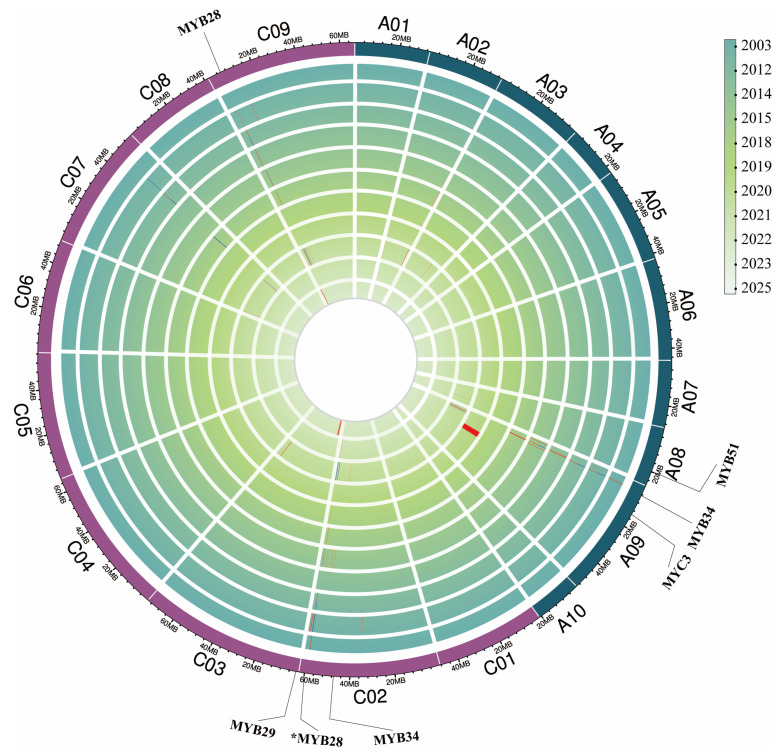
Year-chromosome co-localization map of QTLs associated with GSL content in *Brassica napus*. The outermost track displays the 19 chromosomes of the A and C subgenomes of B. napus (A01–A10, C01–C09), with physical length scales marked in megabases (Mb). The inner concentric tracks correspond sequentially to research years from 2003 (the outermost inner track) to 2025 (the innermost track), where the color gradient scale on the right indicates the year matched to each track (darker colors represent earlier years). The bar segments on each track represent the genomic intervals of GSL-related QTLs identified in the corresponding year’s study, aligned to their physical positions on the chromosomes. The physical positions of key MYB and MYC transcription factors related to GSL biosynthesis (aligned to the Darmor v10 reference genome) are marked outside the chromosomal track via leader lines. The *MYB28* locus marked with an asterisk (*) indicates that this transcription factor is annotated in the *B. napus* cultivar ZS11, while no corresponding annotation is present in the Darmor v10 reference genome used in this study. Full details of all QTL intervals are provided in Supplementary Table S4.

Early mapping studies in *B. napus* consistently identified major QTLs for seed GSL content on chromosomes A09, C02, and C09 (Figure 6), which laid the foundation for initial MAS breeding. With the advent of high-density SNP arrays and whole-genome resequencing, these major loci have been fine-mapped to the gene level: Chao et al. combined QTL mapping with RNA-seq to pinpoint major loci on A09 and C02 that explained up to 40% of the phenotypic variation in seed GSL content [66], whereas Tang et al. identified 113 candidate intervals significantly associated with six distinct GSL metabolites through metabolite GWAS (mGWAS), predicting 187 candidate genes including known regulators *BnaMAM1*, *BnaGGP1*, *BnaSUR1*, and *BnaMYB51*, as well as novel candidates such as *BnaMYB44* and *BnaNAC102* [67]. Beyond seed traits, five stable QTLs associated with GSL levels in floral stems have also been identified, which can be exploited to enhance resistance in young flowering tissues of *Brassica* crops [68].

Meta-analysis of diverse *B. napus* populations has revealed stable QTL hotspots controlling seed GSL content across multiple genetic backgrounds and environments [24,69,70,71,72,73,74,75,76,77,78,79], providing robust targets for molecular breeding. Recent breakthroughs in pan-genomics have further shifted the focus from single-nucleotide polymorphisms (SNPs) to SVs, which are major drivers of phenotypic variation in GSL traits. Zhang et al. [42] constructed a comprehensive reference library of 334,461 SVs from 16 representative *B. napus* morphotypes, and detected 258,865 SVs in 2105 resequenced genomes. Coupling these data with transcriptomes from five tissues, the study uncovered 285,976 SV-expression quantitative trait loci (eQTLs), and identified 726 SV–gene expression–trait associations through joint SV-GWAS and transcriptome-wide association studies, notably demonstrating the pervasive impact of SVs on GSL biosynthesis and transport pathways [42]. Such species-scale SV datasets provide a powerful new resource for gene discovery and precision breeding of GSL profiles.

The integration of GWAS with transcriptomics has accelerated the transition from “QTL intervals” to “causal genes” and functional markers. Beyond the well-known MYB28, MAM1, and AOP2, evolutionary analysis has revealed that allelic variation in side-chain elongation genes and differential expression of upstream regulatory modules contribute significantly to the flavor and resistance divergence among different *B. oleracea* and *B. rapa* varieties [68]. For instance, allelic variation at the *MAM1* locus in *B. oleracea* determines the chain length of aliphatic GSLs, directly influencing both flavor bitterness and pest resistance [68]. A particularly illustrative example of the importance of regulatory variation comes from recent work on progoitrin biosynthesis. Zheng et al. found that a major QTL cluster governing the conversion of gluconapin to progoitrin in *Brassica rapa* was driven by promoter variation in *BrGSL-OHa* rather than a coding sequence mutation. In oil-type *B. rapa*, the *BrGSL-OHa* promoter lacks a MYB transcription factor binding site, rendering it unable to be activated by BrMYB29, thereby blocking progoitrin biosynthesis [80]. This case underscores a critical insight: in QTL/GWAS-based breeding, focusing solely on coding regions is insufficient. Deep functional dissection of non-coding regulatory regions is essential to unlock phenotypic variation and develop truly effective functional markers. In vegetable-type versus oilseed-type *B. rapa*, *BrMYB28.1* and *BrGSL-OH.1* are key determinants of aliphatic GSL profiles, providing functional markers for targeted Chinese cabbage improvement [44].

The identification of these precise loci has facilitated the development of functional markers and GS models for GSL traits. The expression levels of *MYB28* homologs remain attractive major-effect targets for MAS due to their strong positive correlation with aliphatic GSL accumulation [38,81]. For instance, Fu et al. [72] developed genic cleavage markers closely linked to seed GSL content, enabling breeders to select against high-GSL alleles on the C subgenome. However, breeders must navigate “correlated selection effects” and linkage drag within the GSL regulatory network [82]. Using 202 doubled haploid (DH) lines across two seasons, Xu et al. [83] identified loci for GSL and erucic acid that accounted for 83.8% and 89.7% of the phenotypic variation, respectively, and emphasized that GSL content is governed simultaneously by embryo and maternal genetic systems. Resolving these complex genetic systems and breaking unfavorable linkages through marker-assisted backcrossing [76,83] has enabled the optimization of both nutritional quality and plant defense in modern *Brassica* cultivars.

While MAS and GS based on QTL/GWAS have achieved great success in conventional GSL trait improvement, the development of precision genetic engineering and genome editing tools enables more targeted, site-specific modification of GSL profiles, breaking the bottleneck of linkage drag in conventional breeding.

### 7.3. Engineering of Transcriptional Regulatory Modules for Targeted GSL Trait Improvement

As the key determinants of GSL biosynthesis detailed in Chapter 4, R2R3-MYB, MYC, and WRKY transcription factors are the most attractive targets for the genetic engineering of GSL traits, as their modification can achieve systematic regulation of the entire GSL biosynthetic pathway, rather than altering a single metabolic step. The functional conservation of these regulatory modules between *Arabidopsis* and *Brassica* species provides a clear roadmap for targeted engineering.

For oilseed *Brassica* crops, the downregulation or knockout of aliphatic GSL-specific MYB regulators is the most direct strategy to reduce seed antinutritional GSLs. In *B. napus*, targeted knockout of specific *MYB28* homologs significantly reduces harmful seed aliphatic GSLs while retaining essential leaf defense GSLs, effectively mitigating the long-standing “quality-defense trade-off” in rapeseed breeding [18]. Similarly, simultaneous knockout of *BnMYB28* and *BnMYB29* in oilseed rape achieves a significant reduction in total seed aliphatic GSL content and progoitrin levels, providing direct molecular targets for improving the feeding value of rapeseed meal [18]. For vegetable-type crops and resistance breeding, the overexpression of MYB regulators enables targeted enhancement of beneficial or defensive GSLs: overexpression of *MYB28* or *MYB29* markedly enhances aliphatic GSL accumulation, while overexpression of *MYB51* or *MYB122* increases indolic GSL levels, providing practical strategies for generating lines with enhanced insect or disease resistance [29,37].

Beyond MYB key determinants, bHLH family MYC factors, which form core JA-responsive transcriptional complexes with R2R3-MYB proteins, are key targets for engineering inducible GSL defense responses. In both *Arabidopsis* and *Brassica*, stabilizing MYC2/3/4 or enhancing JA signaling amplifies the inducible GSL defense response, thereby improving resistance to herbivores and pathogens [35]. WRKY transcription factors, particularly WRKY33, provide additional targets for pathogen resistance breeding: the WRKY33CYP81F2IGMT module has been shown to mediate immunity to necrotrophic pathogens such as *Alternaria brassicicola* in *Brassica* crops [36], providing novel regulatory nodes for constructing disease-resistant oilseed rape and cabbage varieties. The multigene engineering of these regulatory modules enables both the targeted increase in specific GSL content in planta and the in vitro production of high-value GSL products through heterologous pathway reconstruction.

### 7.4. Genome Editing of Structural and Modification Enzymes for Customized GSL Profiles

Structural genes involved in GSL side-chain elongation, core structure formation, and secondary modification are direct targets for genome editing, enabling the precise redistribution of metabolic flux and customization of GSL profiles. Unlike the global regulation of transcriptional regulators, editing of structural genes enables the modification of specific GSL subtypes without affecting the entire biosynthetic pathway, providing higher precision for trait improvement. The CRISPR-Cas9 system has completed the optimization of the transformation system in the important *Brassica napus* cultivar ZS11, achieving a transgenic positive rate of 94.2% and a mutagenesis frequency of 68.4%, and the edited loci can be stably inherited [84], which provides a mature technical support for the precise genome editing of GSL metabolic structural genes.

For the side-chain elongation and core structure formation stages, genes including *BCAT4*, *MAM1/3*, *IPMDH1*, and *CYP79F1/2* largely determine the total amount and chain length of aliphatic GSLs [13,85]. Genome editing of these genes enables targeted metabolic flux redistribution: repression or knockout of *CYP79F1* can significantly reduce short-chain aliphatic GSLs, mitigating the antinutritional effects of rapeseed meal [86], whereas enhanced expression of *MAM1/3* and *BCAT4* favors increased GSL accumulation for developing highly resistant or functional materials. In *B. napus*, mutants of *BnCYP79F1* show significantly reduced total seed aliphatic GSL levels, with progoitrin decreased by approximately 50%, confirming that knockout of multi-copy major-effect structural genes is an effective route to low-GSL oilseed rape [18].

Side-chain modification enzymes, including AOP2/3, GSL-ALK, and FMO_GS-OX_, are the central targets for tailoring GSL composition, as they determine the allocation of aliphatic GSLs among different modification types with distinct nutritional and defensive values. Extensive analyses of natural germplasm and functional studies have revealed that loss-of-function alleles of *AOP2* promote the accumulation of health-promoting glucoraphanin (GRA), representing a key genetic basis for high-GRA broccoli and cabbage [16,68,87]. The most representative application of this strategy is in Chinese kale (*B. oleracea*), where CRISPR/Cas9-mediated knockout of *BoaAOP2* increases GRA levels by more than tenfold in edible tissues [88]. In *B. juncea*, simultaneous silencing of *GSL-ALK* family genes elevates seed GRA content by 20–40 fold and enhances resistance to *Sclerotinia sclerotiorum*, demonstrating that rational reconstruction of aliphatic modification branches can achieve a “win–win” balance between nutritional quality and disease resistance [37].

**Figure 7:**
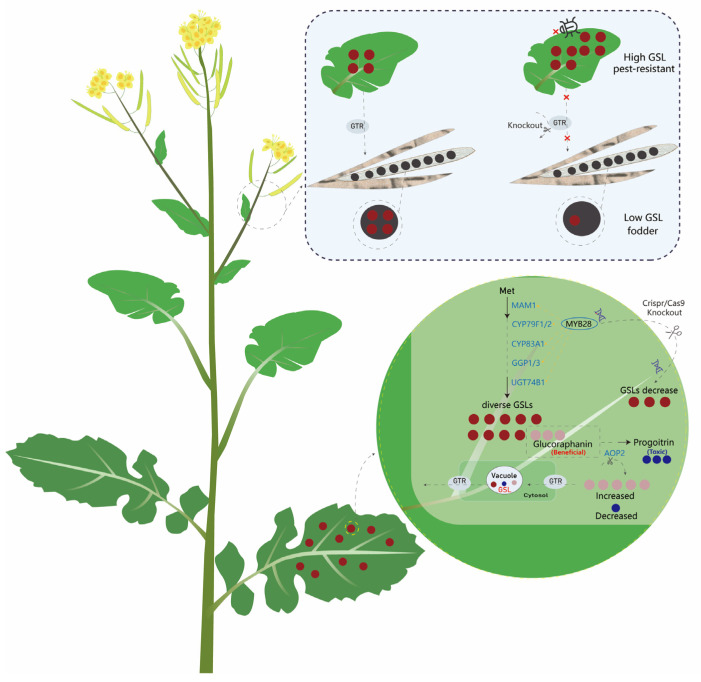
Schematic diagram of gene editing strategies for targeted improvement of GSLs in *Brassica napus*. This figure depicts the metabolic regulatory network of GSL and the CRISPR/Cas9-based gene editing improvement pipeline in *Brassica* plants. Knockout of *GTR* family transporter genes enables differential accumulation of GSLs between leaves and seeds. The upper panel compares two distinct phenotypes: high GSLs accumulation (conferring plant pest resistance) and low GSLs accumulation (feed demand), together with the differential distribution of GSLs in corresponding organs. The lower panel elaborates the intracellular biosynthetic pathway of GSLs: knockout of the transcription factor *MYB28* downregulates total GSLs content in plants, whereas targeted editing of the *AOP2* gene enables directed modulation of GSL profiles, with reduced content of harmful GSLs and elevated content of beneficial GSLs.

Given that oilseed rape and mustard are predominantly polyploid crops, CRISPR/Cas9-mediated multiplex editing using multiple sgRNAs can simultaneously target homologous copies of *AOP2/3*, *CYP79F*, *MAM*, and other structural genes, enabling systematic remodeling of GSL branches without the linkage drag associated with conventional breeding (Figure 7) [16,62].

### 7.5. Emerging Strategies for GSL Trait Optimization

Beyond the direct modification of biosynthetic and regulatory pathways, several emerging strategies have shown great potential for GSL trait improvement, addressing the core bottlenecks in current breeding programs. The inter-organ transport and tissue distribution of GSLs are critical determinants of their breeding applications, as they enable the uncoupling of defensive and nutritional traits. BAT5 participates in the transmembrane transport of chain-elongation substrates between plastids and the cytosol, whereas GTR1/2 mediate GSL loading into the vascular system and their long-distance transport to seeds (Figure 7) [89]. Seed-specific downregulation of *GTR* can markedly reduce seed GSL content while maintaining high levels of defense-related GSLs in leaves, representing a key strategy for “targeted detoxification” without compromising plant resistance. By further combining organ- or tissue-specific promoters, high GSL accumulation can be confined to leaves or roots while reducing bitter GSLs in edible tissues, offering a promising approach to achieving a “high-defense–good-flavor” balance in vegetable-type *Brassica* crops [61,78].

While this strategy holds considerable conceptual promise, its translation into practical breeding applications hinges on a more comprehensive understanding of GSL transport biology. In particular, the feasibility and long-term genetic stability of seed-specific GTR downregulation in polyploid *Brassica* species remain to be rigorously evaluated. The functional redundancy, potential complementation, and as-yet-uncharacterized subfunctionalization among multiple GTR homeologs in these complex genomes represent key variables that must be resolved to achieve predictable phenotypic control. Furthermore, a deeper mechanistic dissection is required to elucidate how in situ biosynthesis and long-distance transport are coordinately regulated to establish the final organ-specific GSL accumulation profiles. Addressing these fundamental questions will be essential to fully harness the potential of transporter engineering for the targeted uncoupling of defense and nutritional traits in *Brassica* crops.

GSL metabolism is also tightly coupled with primary metabolism and environmental signaling pathways, providing additional targets for trait optimization. Glucose can promote indolic GSL biosynthesis without markedly suppressing primary sulfur metabolism, providing a conceptual basis for engineering regulatory circuits involving metabolic sensors and MYB transcription factors [90]. Crosstalk among JA, SA, ethylene, and ABA determines the inducible patterns of different GSL branches under biotic stress [28,35,36], while coordinated regulation of sulfur assimilation pathways enables integrated optimization of “sulfur supply–GSL biosynthesis” to stabilize GSL traits across fluctuating environments [91]. From a synthetic biology perspective, recent studies have attempted to assemble MAM, CYP79, CYP83, and myrosinase into modular metabolic pathways to reconstruct efficient GSL biosynthetic modules in heterologous systems, with the goal of customizing high-value GSLs of different structural types [3]. In parallel, the identification and introduction of thermostable myrosinases into broccoli have been explored to maintain strong GSL hydrolysis capacity at cooking temperatures, providing a novel route for developing “processing-resilient” functional vegetables [92].

## 8. Conclusions and Perspectives

### 8.1. Conclusions

Glucosinolates (GSLs) are central determinants of stress resistance, nutritional quality, and flavor in *Brassica* crops. This review has systematically summarized the chemical classification, tissue-specific distribution, and three-stage biosynthetic pathway of GSLs; dissected the core transcriptional regulatory network dominated by MYB-MYC-WRKY transcription factors; and elucidated how whole-genome duplication (WGD)-driven gene diversification shapes species-specific GSL accumulation patterns. We propose a multi-tiered regulatory model that integrates hormonal signals, nutrient status, and environmental stresses. In the polyploid context of *Brassica*, extensive copy number variation and subfunctionalization of both regulatory factors (e.g., MYB28/29) and structural genes (e.g., AOP2, MAM) confer a regulatory complexity far exceeding that observed in *Arabidopsis thaliana*. Genetic dissection (QTL/GWAS) and breeding practice have confirmed that targeting core nodes of this network (e.g., *BnaC2.MYB28*, *BnaAOP2*) constitutes an effective strategy for GSL trait improvement. Nevertheless, the complete regulatory logic of GSL metabolism in a polyploid background remains to be fully elucidated.

### 8.2. Core Controversies and Critical Knowledge Gaps

Fundamental insights into GSL metabolism have yet to be fully translated into precision breeding, owing to the following unresolved controversies and knowledge gaps.

The mode of WGD-driven functional diversification remains controversial. While traditional views emphasize functional redundancy among homologous genes, emerging evidence reveals a more complex picture. In *Brassica napus*, distinct MYB28 homeologs exhibit tissue-specific regulatory divergence between seeds and leaves. More strikingly, in *Brassica juncea*, the four *Bju.MYB29* copies display opposing effects on indolic GSL regulation—*BjuA03.MYB29* acts as a repressor, whereas *BjuA10.MYB29* functions as an activator. Whether these phenomena represent subfunctionalization (partitioning of ancestral functions) or neofunctionalization (acquisition of novel roles) remains an open question. Furthermore, the molecular drivers underlying such divergence—specific amino acid substitutions or cis-regulatory element variations—have yet to be systematically identified. This controversy directly influences the choice of targets for gene-editing-based breeding.

The molecular basis of tissue-specific regulation and its coupling with transport mechanisms remains largely unresolved. The same core MYB–MYC–WRKY module drives markedly different GSL profiles across tissues: MYB28 serves as a master switch in seeds but contributes minimally in roots, whereas WRKY33 primarily mediates stress-responsive indolic GSL accumulation in leaves. The upstream mechanisms governing such differential regulation—including tissue-specific *cis*-regulatory elements, chromatin accessibility landscapes, and cell-type-specific co-regulators—remain poorly understood. Single-cell studies have confirmed that GSL biosynthesis is confined to specific cell types (e.g., phloem parenchyma cells), raising the additional challenge of coordinated “synthesis-transport” regulation. How GTR/UMAMIT transporters achieve precise source-to-sink allocation of GSLs, and why seed-specific silencing of GTR reduces seed GSLs without affecting foliar levels, are questions whose answers hold the key to resolving the “quality-defense trade-off” breeding bottleneck.

The integrative mechanisms underlying environmental responsiveness and trait stability are insufficiently understood. GSL content is highly sensitive to sulfur nutrition, temperature, and pest/pathogen pressure, yet the genetic and epigenetic basis of its environmental stability remains virtually unexplored. Most GWAS/QTL studies are based on single-environment data and have identified few reliable genotype-by-environment (G × E) interaction loci, hindering the development of broadly adapted cultivars. The molecular switches through which environmental signals are specifically perceived and channeled to distinct GSL branches (e.g., herbivory preferentially inducing aliphatic GSLs, salinity inducing indolic GSLs) remain unidentified. Notably, the role of epigenetic modifications (DNA methylation, histone modifications) as mechanisms of environmental memory and response are only beginning to emerge, and systematic investigation in polyploid *Brassica* is urgently needed.

The translational path from QTL/GWAS to breeding application remains protracted. Although numerous QTLs associated with GSL traits have been identified, the vast majority have not progressed beyond the candidate gene stage. A critical bottleneck is that causal functional variants often reside in non-coding regulatory regions rather than in coding sequences. For instance, in *Brassica rapa*, a deletion of an MYB-binding site in the promoter of *BrGSL-OHa*—rather than variation in the gene body itself—accounts for differences in progoitrin content. In polyploid genomes, accurately distinguishing cis- from trans-regulatory variants amidst extensive linkage disequilibrium of SNPs and structural variants, and developing decisive functional markers, is essential for bridging the gap between “association” and “causality.”

### 8.3. Future Research Directions and Perspectives

Addressing the above bottlenecks requires a shift from descriptive synthesis to mechanistic dissection, focusing on the following directions.

Constructing systematic regulatory maps to decode polyploid complexity. Systematic combinatorial CRISPR knockout of all homeologous copies of key regulators (e.g., MYB28/29) is required to quantify the functional contribution and interaction modes (synergistic, antagonistic, dosage compensation) of each copy in specific tissues and environments. Cross-species comparative genomics will clarify the relative contributions of subfunctionalization versus neofunctionalization.

Deciphering tissue-specific regulation to resolve the “quality-defense” dilemma. ATAC-seq, DAP-seq, and single-cell multi-omics should be employed to map cis-regulatory elements governing tissue-specific expression of core transcription factors across organs and cell types. Concurrent isotope tracing and tissue-specific transporter knockout will resolve the relative contributions of in situ biosynthesis versus long-distance transport, providing a foundation for “low seed GSLs with uncompromised foliar defense.”

Integrating G × E and epigenetic regulation to enhance trait stability. Multi-environment field trials with large populations are needed to develop statistical models that efficiently detect G × E QTLs and clone stability-governing genes. Leveraging *Brassica* epigenomic resources, the dynamics of DNA methylation and histone modifications under sulfur deficiency and low temperature should be systematically investigated to assess the heritability and breeding utility of epialleles.

Bridging the translational path from QTL to cultivar. High-throughput functional validation platforms for non-coding sequences are urgently needed. CRISPR saturation editing of promoter and coding regions within GWAS-identified QTL clusters will pinpoint causal variants. Converting validated functional variants—whether coding or cis-regulatory—into decisive markers and integrating them with genomic selection models will enhance breeding accuracy. Ultimately, a digital predictive model incorporating subgenome-biased expression, homeolog dosage effects, regulatory element effect sizes, and G × E coefficients will enable the shift from “empirical breeding” to “intelligent design breeding.”

Building upon these advances, the inherent limitations of an “all-in-one” ideotype must be acknowledged. Resource trade-offs prevent simultaneous optimization of seed GSLs, vegetative defense, and edible nutrition in a single cultivar. Rather than pursuing “all-around champions,” breeding should pivot toward “specialist champions” tailored to end-use: vegetable types maximizing edible beneficial GSLs, oilseed types minimizing seed anti-nutritional GSLs. More strategically, the environmental sensitivity of GSL metabolism can be exploited to develop specialized cultivars—such as salt-tolerant genotypes with high beneficial GSLs or high-GSL green manure varieties for soil biofumigation. This purpose-driven diversification integrates GSL biochemistry with sustainable agriculture, expanding the application spectrum of *Brassica* crops. Through systematic research spanning mechanistic dissection to technological innovation, we can transcend the inherent “quality-defense-environmental adaptation” trade-off, shifting from “all-around champions” toward purpose-driven “specialist champions,” ultimately advancing the precision and sustainability of the *Brassica* industry.

## Data Availability

No new data were created or analyzed in this study. Data sharing is not applicable to this article.

## Supplementary Materials

The following supporting information can be downloaded at: https://www.mdpi.com/article/10.3390/ijms27093725/s1.
