# Supplementary material for: Glucosinolates in Brassica Species: Biosynthesis, Regulation, and Molecular Breeding
Source: Int J Mol Sci. 2026 Apr 22;27(9):3725. doi: 10.3390/ijms27093725 (PMC13164505; doi:10.3390/ijms27093725)
Supplement: Supplementary file 1 [file ijms-27-03725-s001.zip › Supplementary Figure.pdf]

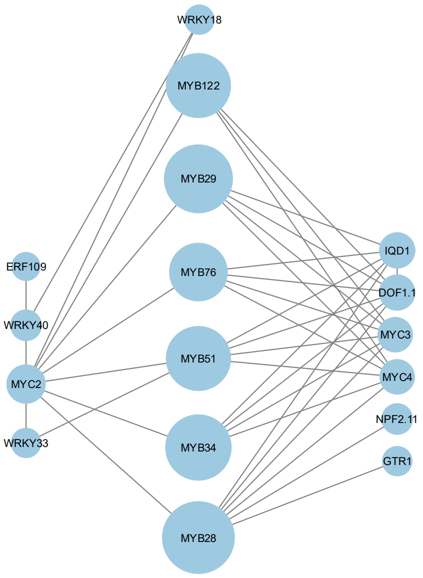

Supplementary Figure S1. PPI network of transcription factors in glucosinolate biosynthesis

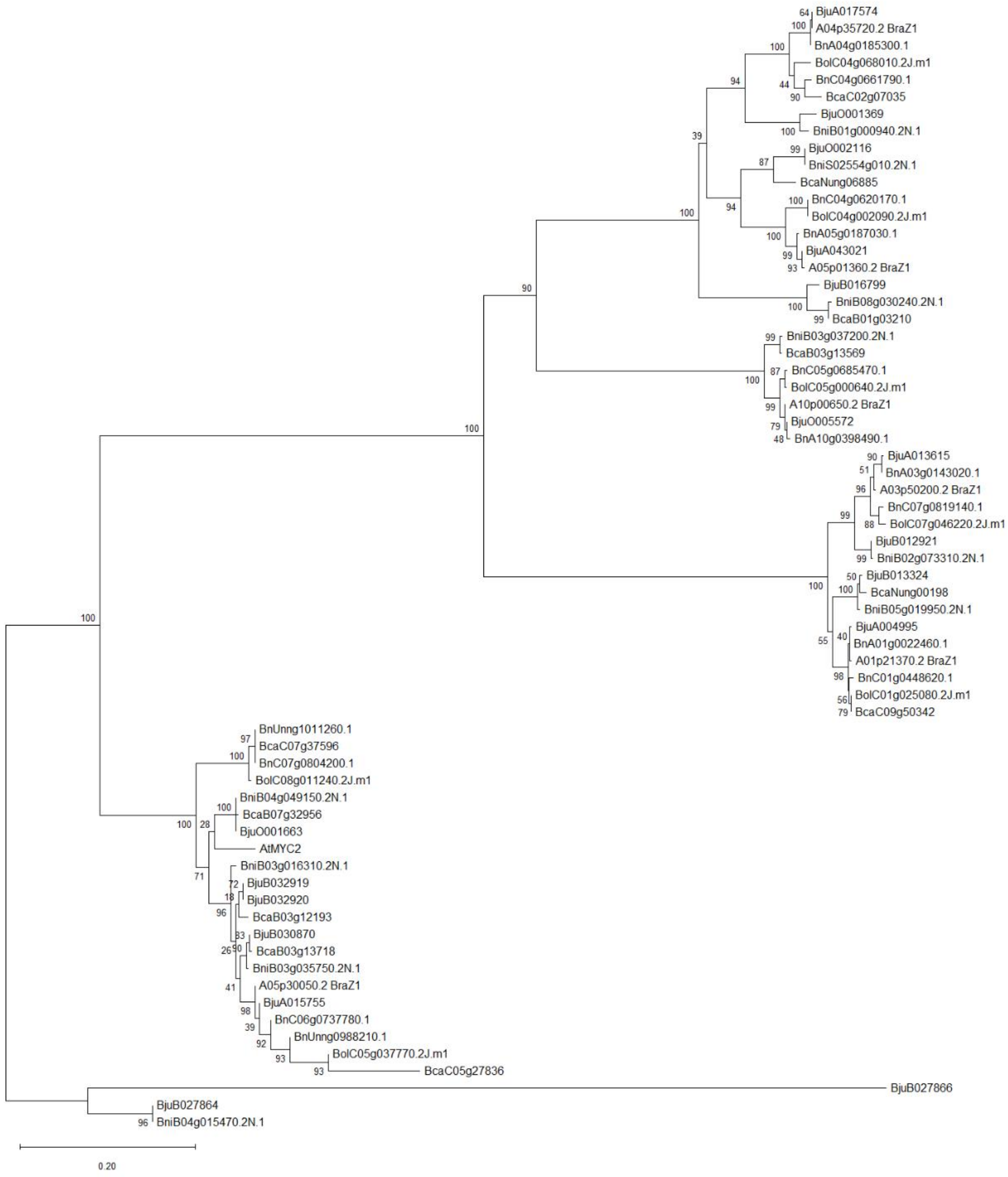

Supplementary Figure S2. Phylogenetic tree of transcription factor MYC2.

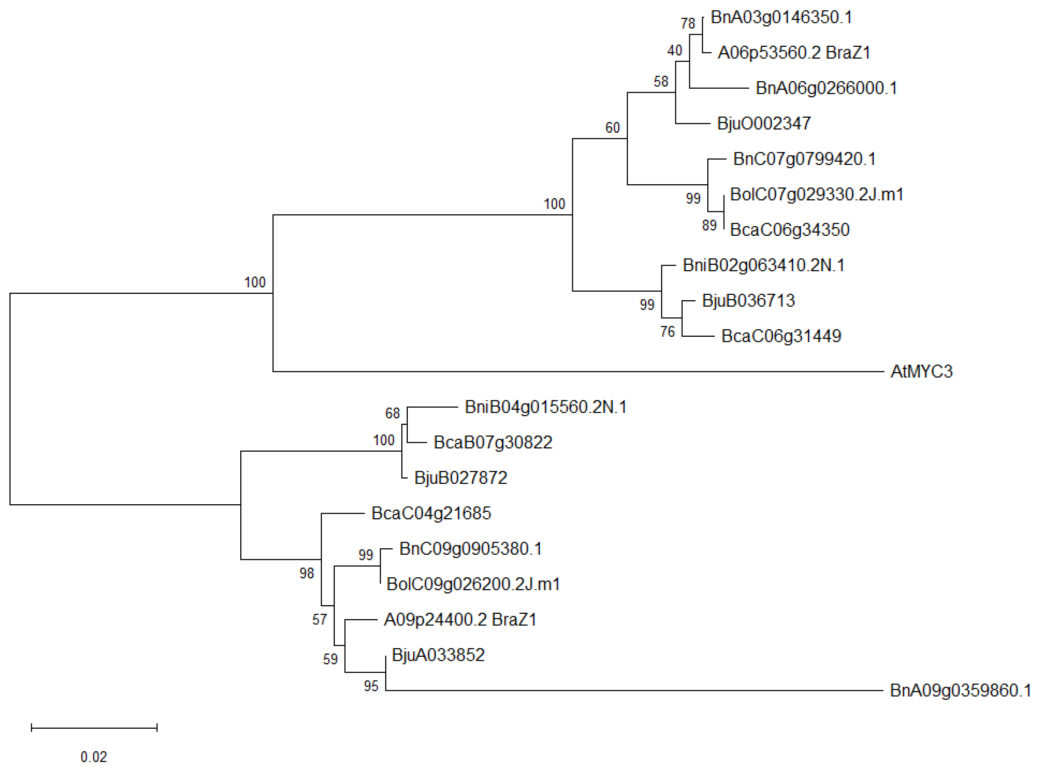

Supplementary Figure S3. Phylogenetic tree of transcription factor MYC3.

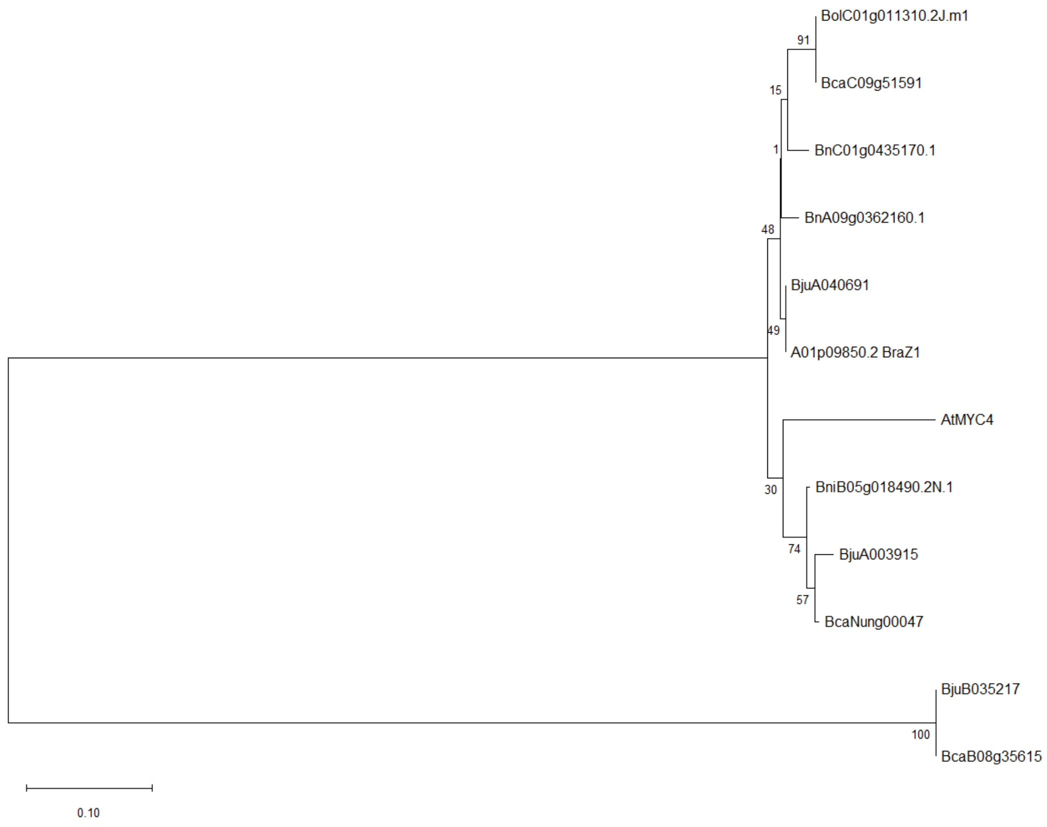

Supplementary Figure S4. Phylogenetic tree of transcription factor MYC4.

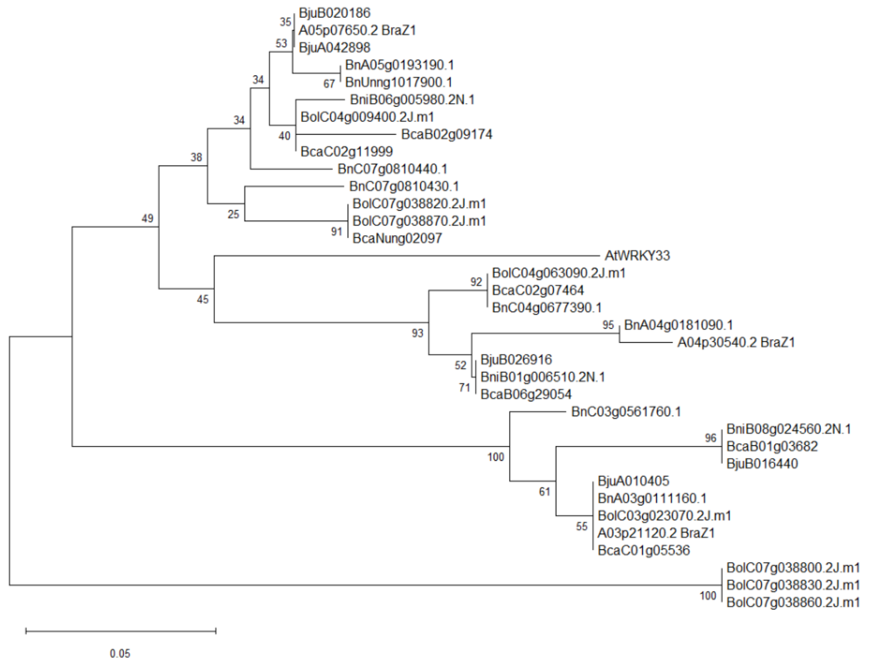

Supplementary Figure S5. Phylogenetic tree of transcription factor WRKY33
